# Inhibition of CCL7 derived from Mo-MDSCs prevents metastatic progression from latency in colorectal cancer

**DOI:** 10.1038/s41419-021-03698-5

**Published:** 2021-05-13

**Authors:** Xiaoli Ren, Jianbiao Xiao, Wanning Zhang, Feifei Wang, Yongrong Yan, Xuehui Wu, Zhicheng Zeng, Yumei He, Wei Yang, Wangjun Liao, Yanqing Ding, Li Liang

**Affiliations:** 1 Department of Pathology, Nanfang Hospital and Basic Medical College, Southern Medical University, Guangzhou, 510515, Guangdong Province People’s Republic of China; 2 Department of Pathology, The Affiliated Hospital of Southwest Medical University, Luzhou, 646000, Sichuan Province People’s Republic of China; 3 Guangdong Province Key Laboratory of Molecular Tumor Pathology, Guangzhou, 510515, Guangdong Province People’s Republic of China; 4 Department of Immunology, Southern Medical University, Guangzhou, 510515, Guangdong Province People’s Republic of China; 5 Department of Oncology, Nanfang Hospital, Southern Medical University, Guangzhou, 510515, Guangdong Province People’s Republic of China

**Keywords:** Cancer models, Colorectal cancer, Metastasis, Tumour immunology

## Abstract

In colorectal cancer (CRC), overt metastases often appear after years of latency. But the signals that cause micro-metastatic cells to remain indolent, thereby enabling them to survive for extended periods of time, are unclear. Immunofluorescence and co-immunoprecipitation assays were used to explore the co-localization of CCL7 and CCR2. Immunohistochemical (IHC) assays were employed to detect the characters of metastatic HT29 cells in mice liver. Flow cytometry assays were performed to detect the immune cells. Bruberin vivo MS FX Pro Imager was used to observe the liver metastasis of CRC in mice. Quantitative real-time PCR (qRT-PCR) and western blot were employed to detect the expressions of related proteins. Trace RNA sequencing was employed to identify differentially expressed genes in MDSCs from liver micro-M and macro-M of CRC in mice. Here, we firstly constructed the vitro dormant cell models and metastatic dormant animal models of colorectal cancer. Then we found that myeloid-derived suppressor cells (MDSCs) were increased significantly from liver micro-metastases to macro-metastases of CRC in mice. Moreover, monocytic MDSCs (Mo-MDSC) significantly promoted the dormant activation of micro-metastatic cells compared to polymorphonuclear MDSCs (PMN-MDSC). Mechanistically, CCL7 secreted by Mo-MDSCs bound with membrane protein CCR2 of micro-metastatic cells and then stimulated the JAK/STAT3 pathway to activate the dormant cells. Low-dose administration of CCL7 and MDSCs inhibitors in vivo could significantly maintain the CRC metastatic cells dormant status for a long time to reduce metastasis or recurrence after radical operation. Clinically, the level of CCL7 in blood was positively related to the number of Mo-MDSCs in CCR patients, and highly linked with the short-time recurrence and distant metastasis. CCL7 secreted by Mo-MDSCs plays an important role in initiating the outgrowth of metastatic latent CRC cells. Inhibition of CCL7 might provide a potential therapeutic strategy for the prevention of metastasis recurrence.

## Background

Increasing evidence demonstrates that metastasis, the spread of tumor cells, can occur already at the early stages of primary tumor progression^1,2^. Instead of fast growth to visible metastases, these cells will be latency state (non-growing or slow-growing status) for a long time^3,4^. Colorectal cancer (CRC) patients often develop distant recurrence years after surgical removal of the primary tumor. Understanding the mechanism of metastatic recurrence after latency is crucial for improving the cure rate for CRC. Evidence shows that cancer can remain latency either as quiescent cells (cellular dormancy) or as indolent small clusters that maintain balanced proliferation and death (tumor mass dormancy)^5^. The immune system is the main involvement in maintaining cancer cells in dormancy or activating them for fast growth. The balance between immunosuppressive cells, especially myeloid-derived suppressor cells (MDSC) and Treg cells, and antitumor immunity cells such as cytotoxic T lymphocyte (CTLs) and Natural kill (NK) cells mainly maintain an equilibrium state. MDSC are a heterogeneous population of pathologically activated myeloid precursors, play a major role in the suppression of antitumor immunity and tumor metastasis^6^. Initial studies reported that the number of MDSC in peripheral blood was positively correlated with cancer stage and tumor burden in colorectal, breast, bladder, thyroid, and non-small cell lung cancers^7–11^. In melanoma and breast cancer, the numbers PMN- and M-MDSC correlate with tumor stage and metastasis^12^. Mechanistically, matrix metalloproteinase (MMP) such as MMP9 secreted by MDSCs can destroy the extracellular matrix around the tumor tissue and promote metastasis^13–15^. In addition, MDSCs induce epithelial to mesenchymal transition (EMT) of cancer cells through signaling pathways such as TGF-β and EGF, which is conducive to the spread of cancer cells^16,17^. Exosomal miR-126a released from MDSC induced by DOX treatment promotes lung metastasis^18^. Although the clinical role of MDSC has emerged in recent years, the role and alteration of MDSCs during the “tumor dormant stage” are still not well understood.

In this study, we aim to investigate whether MDSCs have an effect on the activation of dormant metastatic CRC cells. We reveal that Mo-MDSCs can secrete chemokine (C–C motif) ligand 7 (CCL7) to activate “dormant cells” for metastatic outgrowth through JAK/STAT3 pathway. Most importantly, CCL7 might be a potential therapeutic target for the prevention of CRC metastasis progression from indolent micrometastasis to overt macrometatasis.

## Materials and methods

### Cell culture and transfection Human

CRC cell lines SW480, SW620, HCT116, RKO, HT29, and LOVO were purchased from American Type Culture Collection (ATCC, Manassas, VA) and authenticated in 2017 and confirmed mycoplasma negative by the service provider. All cell lines were cultured in 1640 medium (GIBCO, Gaithersburg, MD, USA) with 10% fetal bovine serum (HyClone, Logan, USA) at 37 °C under 5% CO_2_. For depletion of CCL7 and CCR2, two vectors carrying two human siRNA1 or siRNA2 or siRNA3 toward CCL7 or CCR2 (Ribobio Co. Guangzhou, China) were transfected into MDSCs or vitro-induced-dormant cells. The sequences were shown in Supplementary Table 3.

### ELIAS assay

Levels of 9 cytokines including MMP1 (ELISAGenie, #MOFI00978, London, UK)、CXCL4 (Solarbio, #SEKM-0047, Beijing, China), CXCL5 (Abnova, # KA1799), CCL4 (Abnova, #KA220), CCL7(Abnova, #KA2199), CXCL1 (Abnova, #KA0553), G-CSF (Solarbio, #SEKM-0040) and CCL3 (Abnova, #KA1804) in MDSCs supernatant were measured by duplicated determination with a commercially available ELISA method. Intraobserver variability of the measurements was also assessed and the mean intra-assay coefficients of variance were all <4.5%.

### Cell proliferation assay

1 × 10^3^ cells were seeded into 96-well plates. The number of viable cells was determined by cell counting kit-8 (CCK-8) (Dojindo, Kumamoto, Japan) for 6 days. Briefly, 10 mL CCK-8 solution was added, and absorbance at 490 nm was measured after 2 h of incubation at 37 °C. For plate colony assay, 800 cells were seeded into 6-well plates and cultured at 5% CO_2_, 37 °C for 2 weeks. Then we counted the number of colonies (each colony >50 cells) that were stained with hematoxylin. Each cell group was plated in 3 duplicate wells.

### Immunohistochemistry (IHC)

In brief, paraffin-embedded specimens were cut into 4 mm sections. The sections were deparaffinized with xylenes and rehydrated. Sections were submerged into EDTA antigenic retrieval buffer and microwaved for antigenic retrieval. The sections were treated with 3% hydrogen peroxide in methanol to quench the endogenous peroxidase activity, followed by incubation with 1% bovine serum albumin to block the nonspecific binding. Antibodies against CK (1:300), Ki67 (1:1000), CD44 (1:200), Vimentin (1:1000), c-myc (1:200) were incubated with the sections overnight at 4 °C, respectively. After incubation with secondary antibody, the visualization signal was developed with 3,3′-diaminobenzidine tetrachloride (DAB). The stained tissue sections were reviewed and scored separately by two pathologists blinded to the clinical parameters.

### RNA extraction and real-time RT–PCR

Total RNA was extracted using TRIzol reagent (Invitrogen, Carlsbad, CA, USA) and cDNA was synthesized by using an access reverse transcription system (Promega, Madison, WI, USA). The Real-time Q-PCR primers were shown in Supplementary Table 1. Real-time Q-PCR primers. The first-strand cDNA was synthesized using the PrimeScript RT reagent Kit (TaKaRa, Dalian, China). Real-time PCR was performed using SYBR Premix Ex Taq II (TaKaRa, Dalian, China) and measured in a LightCycler 480 system (Roche, Basel, Switzerland). Expression of GAPDH was used as an internal control. All the reactions were run in triplicate.

### Western blotting

Protein lysates were prepared, subjected to SDS/PAGE, transferred onto PVDF membranes, and blotted according to standard methods using polyclonal or monoclonal antibodies. A mouse monoclonal anti-GAPDH or α-Tublin antibody was used as inner control to confirm equal loading of proteins. More information about antibodies for western blotting were shown in Supplementary Table 2.

### Statistical analysis

The investigator was blinded absolutely to the group allocation during all the experiments or when assessing the outcome, including in the animal assays. In order to have sufficient mice for statistical analysis, we used 8 mice for related in vivo experiments of each group. For correlation analysis, we collected 30 samples of serum of CRC patients in each group. The in vitro assays were repeated at least three independent experiments. The correlation of the level of CCL7 in serum with the number of Mo-MDSCs in the blood of CRC patients or recurrence time was analyzed by Pearson test. Survival analyses were performed according to the Kaplan–Meier method and compared by the log-rank test. Before the analysis of variance, the Levene test was used for variance. Homogeneity test, LSD method was used for multiple comparisons of variances; Dunnett’s 3 method was used for multiple comparisons when variance was not used; Cell proliferation and Edu assays were tested using one-way ANOVA. All statistical analyses were analyzed using the SPSS 20.0 software and *P* < 0.05 was considered significant.

### In vitro cell scratch assay

To quantify cell migration, a scratch was made down the center of each well in a 24-well plate using a p1000 pipette tip at 48 h after transfection. Along the scratch line, the cells were washed away and replaced with a serum-free culture medium. And then we took pictures every 24 h for 5 days. The area between the parallel cell edges was measured at each time point. For each well, three different fields along the scratch were analyzed in triplicate. Cell motility was calculated as the percentage of the cell migration distance with respect to the initial scratch distance.

### In vitro chemotherapy dormancy model of CRC cells

HT29 cells were cultured in a 96-well plate. Oxaliplatin and 5-FU were used to treat HT29 cells at a concentration of 1, 5, 10, and 20 μm/mL for 48 h. After drug treatment, cell proliferation was performed by CCK-8 and EdU assays. The concentration of drug with effectively reduced cell proliferation and good cell viability was selected as the appropriate one for chemotherapy. Following drug treatment, HT29 cells were treated with CCL7 (20 ng/mL), Bindarit (0.15 μM), or INCB3284 (4 nM), and the proliferation of cells was detected by CCK-8 and EdU.

### Construction of liver metastatic dormancy model of CRC in nude mice

2 × 10^6^ HT29 cells were mixed with 3 mg/mL Matrigel matrix were injected into the cecum of nude mice. Mice were sacrificed daily for the observation of the growth of liver metastatic tumors. The growth curves of liver micro-metastases in mice were drawn according to the size of the tumor under the microscope.

### Construction of a tracer model for liver metastatic dormancy of CRC in C57 mice

Four to six months C57BL/6 female mice were allocated randomly to each group and There are 8 mice in each group. 1 × 10^7^ CMT-93 cells transfected with luciferase were mixed with 3 mg/mL matrigel matrix and injected into the cecum of C57BL/6 mice. Caliper IVIS Lumina II was used to observe the tumor formation in the cecum and liver. Before visible liver metastases formed (7 days after injection), the primary tumor in the cecum was surgically removed. Then the mice were treated with Bindarit (5 mg/kg) or Entinostat (10 mg/kg) by gavage twice a week for 1 month. Bruberin vivo MS FX Pro Imager (Bruker, Billerica, MA, USA) was used to observe the liver metastasis of CRC. The growth curve of liver metastases of CRC in mice was drawn according to the fluorescence value of metastatic tumors.

### Immunofluorescence

Cells in culture dishes were fixed with 4% PFS for 5 min at room temperature (RT), followed by washing with PBS. Then they were permeabilized with 0.25% Triton X-100 in PBS for 5 min, washed again with PBS before being blocked with Goat Serum for 30 min. After that, cells were incubated with primary antibodies (rabbit anti-CCL7, 1:200 and mouse anti-CCR2, 1:100) diluted in blocking buffer for 1 h at RT in a dark humid chamber. After washing with PBS, they were incubated with diluted secondary antibody for 1 h at RT before being counterstained with DAPI.

### Isolation of tumor cells from macro- and micro-metastases

The livers of nude mice (5 samples in the macro-metastases group and 15 samples in the micro-metastases group) were ground and filtered through a 70-μm cell strainer. To eliminate the erythrocytes, single-cell suspensions were treated with a hypotonic lysis buffer. Tumor cells were stained for 30 min at 4 °C with appropriate dilutions of Alexa Fluor® 647 Mouse anti-human cytokeratin antibody (#563614, BD). The stained cells were acquired on a FACS Canto II (BD Biosciences) and at least 10^5^ cells were collected in a 1.5 mL pipe for western blotting.

### Analysis of MDSCs in CRC patients’ peripheral blood

The peripheral blood samples of CRC patients were collected in anticoagulation tubes from Dept. General Surgery of Nanfang hospital, Southern Medical University. Leukocytes from CRC patients’ peripheral blood were obtained by Isolated leucocytes from human peripheral blood protocol (Solarbio, P8670). Fresh anticoagulant blood was mixed with the diluent in the ratio of 1:1, and then carefully added to the liquid surface of the separation solution. The mixture was centrifuged at 500 × *g* for 25 min, and the second layer of cells, the third layer of separating solution, and the fourth layer of red blood cells were collected and put into a tube containing 10 mL of cell washing solution. All layers of cells were mixed well and centrifuged at 500 × *g* for 30 min. After the red blood cells were lysed with lysate of red blood cells, the precipitated cells are considered white blood cells. The single-cell suspensions were stained for 30 min at 4 °C with appropriate dilutions of various combinations of the following fluorochrome-conjugated antibodies: anti-human CD11b-APC (#301309, Biolegend), anti-human CD33-APC/Cy7 (#366614, BD Biolegend), anti-human CD14-FITC (#301804, Biolegend), anti-human CD15-PE/Cy7 (#323030, Biolegend), anti-human HLA-DR-BV510 (#563083, BD). The stained cells were acquired on a FACS Canto II (BD Biosciences) and the data were analyzed by using FACS Diva software (BD Biosciences) and Flow Jo 7.6.1 software (Treestar).

### Analysis of immune cells from the liver metastases of CRC in mice

The livers of C57BL/6 mice were ground and filtered through a 70-μm cell strainer. To eliminate the erythrocytes, single-cell suspensions were treated with a hypotonic lysis buffer. The single-cell suspensions were stained for 30 min at 4 °C with appropriate dilutions of various combinations of the following fluorochrome-conjugated antibodies: anti-mouse CD11b-APC (#101212, Biolegend), anti-mouse CD45-PE/Cy7 (#103114, Biolegend), anti-mouse Ly6G-APC/Cy7 (clone RB6-8C5, Abnova), anti-Ly6C-PE (#128008, Biolegend), anti-mouse CD3-BV510 (#100233, Biolegend), anti-mouse CD4-FITC (#100510, Biolegend), anti-mouse NK1.1- BV605 (#563220, BD Biosciences), anti-mouse F4/80-APC/Cy5.5 (#123118, Biolegend), anti-mouse CD8-PE/Cy5.5 (clone 53-6.7, Abnova), anti-CD11C-BV421 (#371511, Biolegend) and anti-mouse Gr-1-APC (#108411, Biolegend). The stained cells were acquired on a FACS Canto II (BD Biosciences) and the data were analyzed by using FACS Diva software (BD Biosciences) and Flow Jo 7.6.1 software (Treestar).

### Co-immunoprecipitation (CoIP)

The cell lysate was incubated 2 h at 4 °C with IgG and protein A + G Agarose to get rid of unspecific binding. CCR2 and CCL7 antibodies were then added at 4 °C overnight. The protein A/G-agarose was collected by centrifugation. Immuno-precipitated proteins were analyzed by SDS-PAGE (10%, Minigel) at 100 V for 1.5 h. CCR2 and CCL7 antibodies were diluted, respectively, and incubated with membranes at 4 °C overnight. The secondary antibodies were then incubated for 1 h at RT. Protein bands were visualized using enhanced chemiluminescence (PerkinElmer Life Sciences). The experiments were repeated three times.

### Surface and intracellular flow cytometry staining

For all in vitro assays, the spleen was excised and a cell suspension was obtained. CD8^+^ T cells were isolated using Dynabeads® Mouse T-Activator CD3/CD28 for T-Cell magnetic beads (Invitrogen, American). The purity of CD8^+^ T cells was >95%, as determined by FACS analysis. Purified CD8^+^ T cells were stimulated with solid-phase anti-CD3 antibody (0.2 μg/mL) and anti-CD28 antibody (2 μg/mL) and Con A (1 μg/mL) for 3 days, and then co-cultured with MDSCs cells for 24 h. And then CD8^+^ T cells were re-stimulated in vitro for 4 h at 37 °C with PMA (50 ng/mL; Sigma-Aldrich) and Ionomycin (1 μg/mL; Sigma-Aldrich) in the presence of 1 μg/mL Brefeldin A. Cells were stained for the following surface markers: CD3 (#100233, Biolegend), CD8 (clone 53-6.7) for 30 min and fixed in flow cytometry buffer plus 2% PFA. Cells were then permeabilized for 5 min with flow cytometry buffer containing 2% saponin and were stained for 15 min at 20 °C with fluorescence-conjugated FITC anti-IFNγ (#505806, Biolegend) in flow cytometry buffer and 1% saponin. Cells were kept in flow cytometry buffer and 1% PFA before analysis.

### Trace RNA sequencing

The RNA sequencing experiment was carried out at Gene Denovo scientific company (Guangzhou, China). Low-input samples (<10^6^ cells) were collected and Total RNA was extracted using PureLink™ RNA Mini Kit (#12183018A, Invitrogen, USA) according to the manufacturer’s protocol. RNA quality was assessed on an Agilent 2100 Bioanalyzer (Agilent Technologies, Palo Alto, CA, USA) and checked using RNase-free agarose gel electrophoresis. After total RNA was extracted, eukaryotic mRNA was enriched by Oligo(dT) beads, while prokaryotic mRNA was enriched by removing rRNA by Ribo-ZeroTM Magnetic Kit (Epicentre, Madison, WI, USA). Then the enriched mRNA was fragmented into short fragments using fragmentation buffer and reverse transcripted into cDNA with random primers. Second-strand cDNA was synthesized by DNA polymerase I, RNase H, dNTP, and buffer. Then the cDNA fragments were purified with QiaQuick PCR extraction kit (Qiagen, Venlo, The Netherlands), end-repaired, poly(A) added, and ligated to Illumina sequencing adapters. The ligation products were size selected by agarose gel electrophoresis, PCR amplified and sequenced using Illumina HiSeq2500 by Gene Denovo Biotechnology Co. (Guangzhou, China).

### Study approval

All protocols for animal studies were reviewed and approved by the Institutional Animal Care and Use Committee of Southern Medical University in accordance with the NIH Guide for the Care and Use of Laboratory Animals (National Academies Press 2011) and the Animal Welfare Act. Approval for experiments on human tissues was obtained from Southern Medical University Ethics Committee for Human Genome Research. Written informed consent was obtained for all study participants.

## Results

### MDSCs were increased rapidly from liver micrometastasis (micro-M) to macrometastasis (macro-M) of CRC in mice

**Fig. 1:**
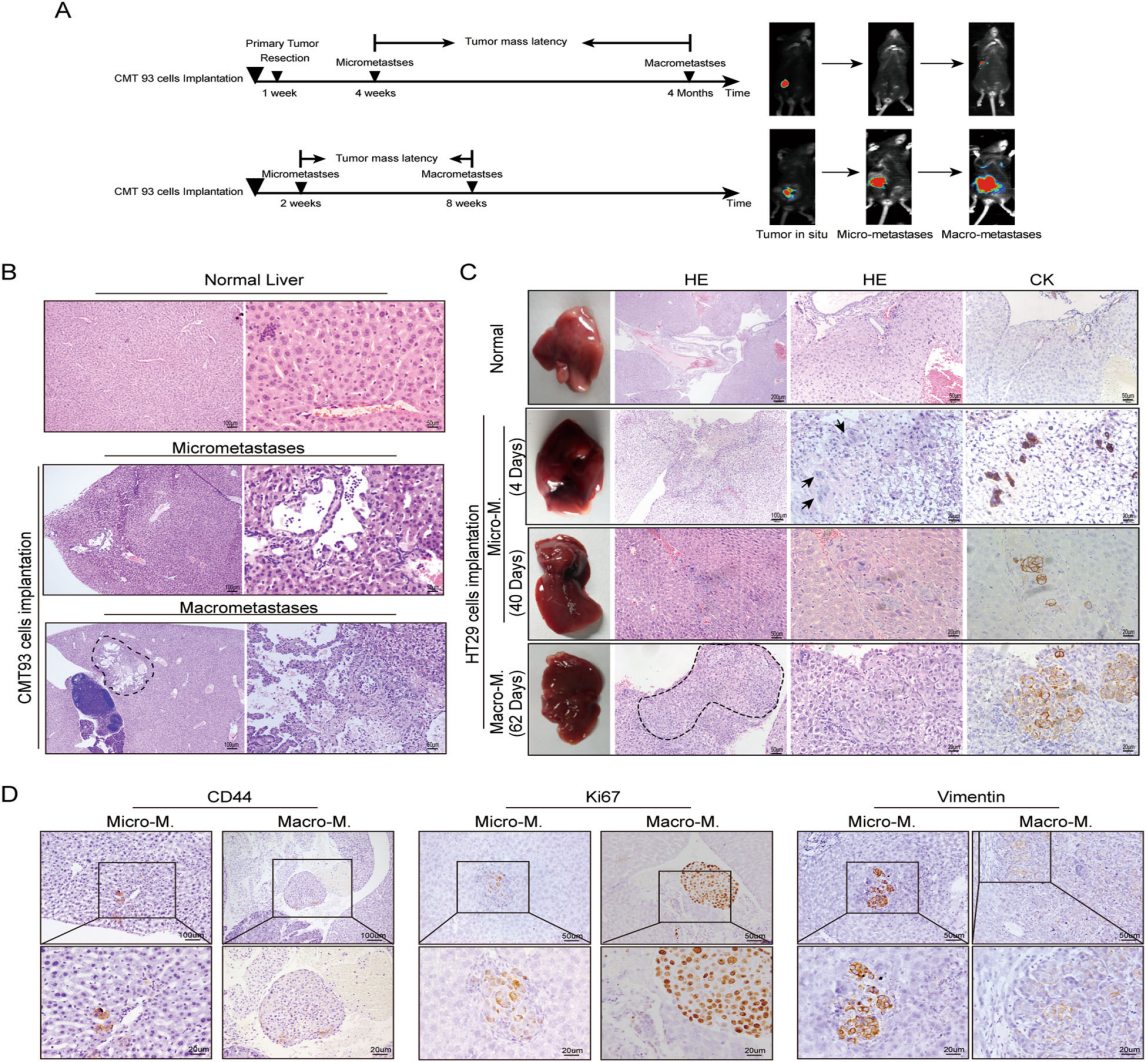
Construction of CRC metastatic dormant models in mice. **A** A brief sketch of two animal models of CRC metastasis in C57 mice. **B** Representative images of liver metastases in different metastatic stages. **C** Observation of liver metastasis of HT29 cells after injected into spleen of nude mice by HE and immunohistochemistry stained with CK7. **D** The immunohistochemistry stain of CD44 or Ki67 or Vimentin in liver metastasis of nude mice injected with HT29 cells.

Firstly, we observed the dormant stage in metastatic models of CRC in C57BL/6 mice with immunity. CMT-93-luciferase cells were implanted into cecum serosa of C57BL/6 mice and the primary tumor was removed 1 week later or not. Primary tumors or liver metastases were observed by Bruber In Vivo MS FX Pro Imager once a week for 4 months (Fig. 1A). The imaging results showed that micro-M appeared in the 4th week, lasting for 3.6 months in the primary tumor resection group. However, in the unresected group, micro-M occurred in the 2nd week, lasting for 6 weeks (Fig. 1A). Microscopically, scattered tumor cells or small tumor cell clusters were observed in the liver micro-M in both groups (Fig. 1B). We then constructed a metastatic dormancy model in BALB/c nude mice. Since evidence shows that EMT and stem cell-like traits are intertwined processes in the transition of dormant disseminated tumor cells to metastatic outgrowth^19^, we detected the expression of markers associated with proliferation, stemness, and EMT in six CRC cell lines and found that HT29 cell line not only lowly expressed cyclin D1, c-Myc, vimentin, but also highly expressed E-cadherin, CD133, SOX2 and Lgr5 (Supplementary Fig. S1A–I). In addition, HT29 is a low-grade CRC cell line (grade II) with low metastatic potential. Therefore, we identified that the HT29 cell line had dormancy characteristics. We injected HT29 cells into the spleen of nude mice and observed the liver micro-M and macro-M through imaging and microscope. Liver micro-M existed from the 4th day to the 40th day. Liver macro-M could be observed after the 40th day (Fig. 1C and Supplementary Fig. S1J). Micro-metastatic cells in BALB/c nude mice presented the higher expression of CD44, Vimentin, and CD133, and lower expression of Ki67, c-Myc, E-cadherin than macrometastatic cells by IHC and immunofluorescence (Fig. 1D and Supplementary Fig. S1K, L). Meanwhile, we isolated the CRC cells from liver micro-M and macro-M in C57BL/6 mice by flow cytometry and detected the expression of the above makers by Western blot. The results of Western blot were consistent with those of IHC and IF (Supplementary Fig. S1M). The data show that liver micro-M of CRC is characterized by dormancy.

**Fig. 2:**
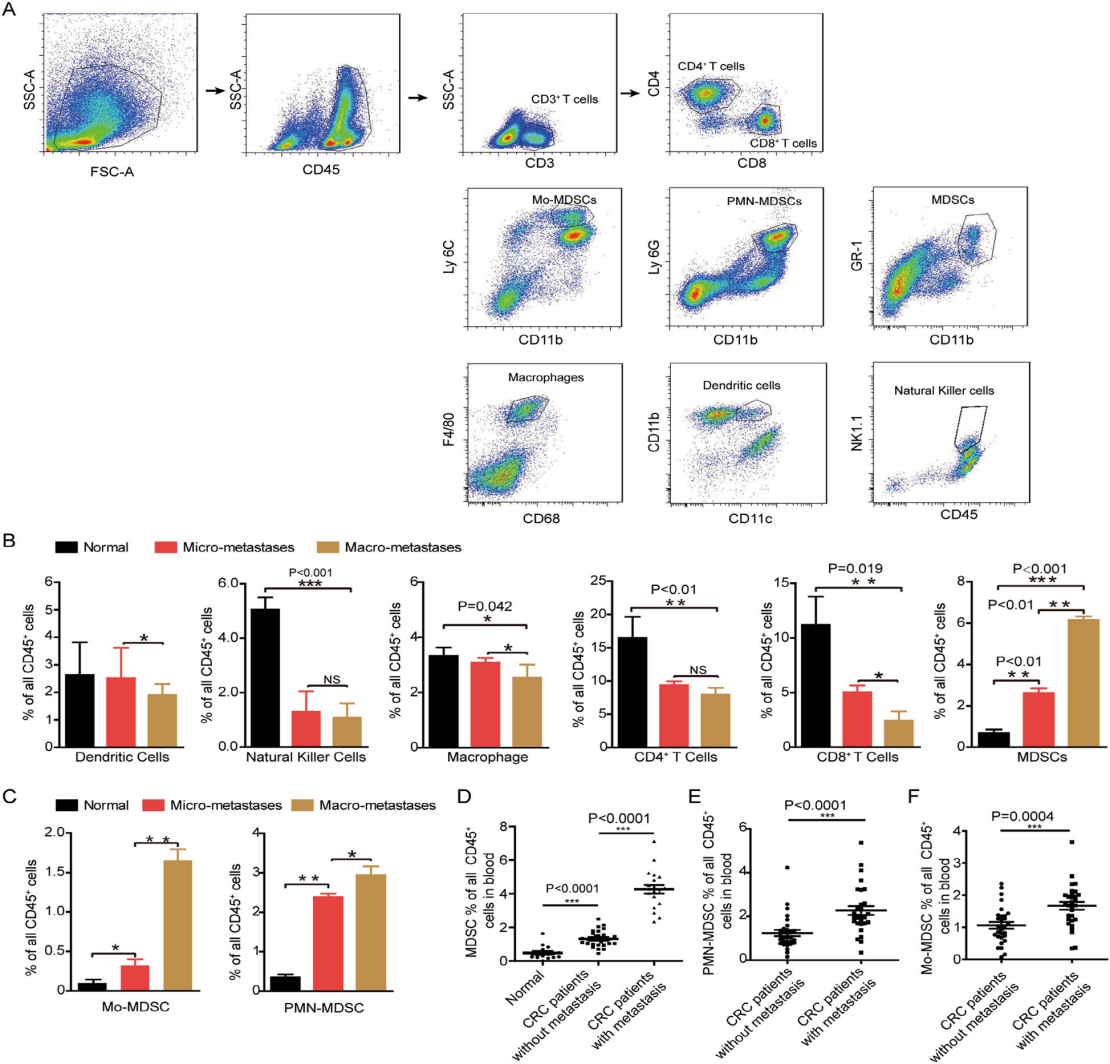
MDSCs constantly accumulated in the liver during CRC process. **A** Diagrams of immune cells by flow cytometry sorting. CD45^+^ cells were first sorted and were stained with CD3 and CD4 antibodies; CD8^+^ T cells were stained with CD3 and CD8 antibodies; dendritic cells were stained with CD11b and CD11c antibodies; macrophages were stained with F4/80 and CD11b antibodies; natural killer cells were stained with NK1.1 antibodies; MDSCs were stained with CD11b, Gr-1 or Ly6G or Ly6C antibodies. **B** Analysis of the numbers of immune cells in CRC liver metastases. **C** Analysis of the numbers of Mo-MDSCs or PMN-MDSCs in CRC liver metastases. **P < 0.01, ***P < 0.001. **D**–**F** Analyses of the MDSCs percent in CRC patients’ blood. Normal (*n* = 20), CRC with metastasis (*n* = 30), CRC without metastasis (*n* = 30). Error bars represent mean ± SD from at least three independent experiments. **P < 0.01, ***P < 0.001.

Immune system plays a vital role in keeping cancer cells dormant or activating. To test what kind of immune cells dominates the transition from dormant stage to metastatic outgrowth, we analyzed the immune profiles between liver micro-M and macro-M in C57BL/6 mice. The results of flow cytometry showed that from micro-M to macro-M, the number of dendritic cells, CD8^+^ T cells, and macrophages were decreased (Fig. 2B, *P* < 0.05), while MDSCs were obviously accumulated in the liver (Fig. 2B, *P* < 0.01). And the increase of Mo-MDSCs was more obvious than PMN-MDSC from liver micro-M to macro-M (Fig. 2C). In addition, the number of MDSCs in peripheral blood of CRC patients with metastasis was higher than that of patients without metastasis (Fig. 2D). The increase of Mo-MDSCs and PMN-MDSCs in the blood of CRC patients was both associated with metastasis (Fig. 2D–F). The above results show that in the process of micrometastasis to micrometastasis, MDSC is one of the fastest-growing immune cells in mice.

### MDSCs activate dormant CRC cells to outgrowth in vitro and in vivo

**Fig. 3:**
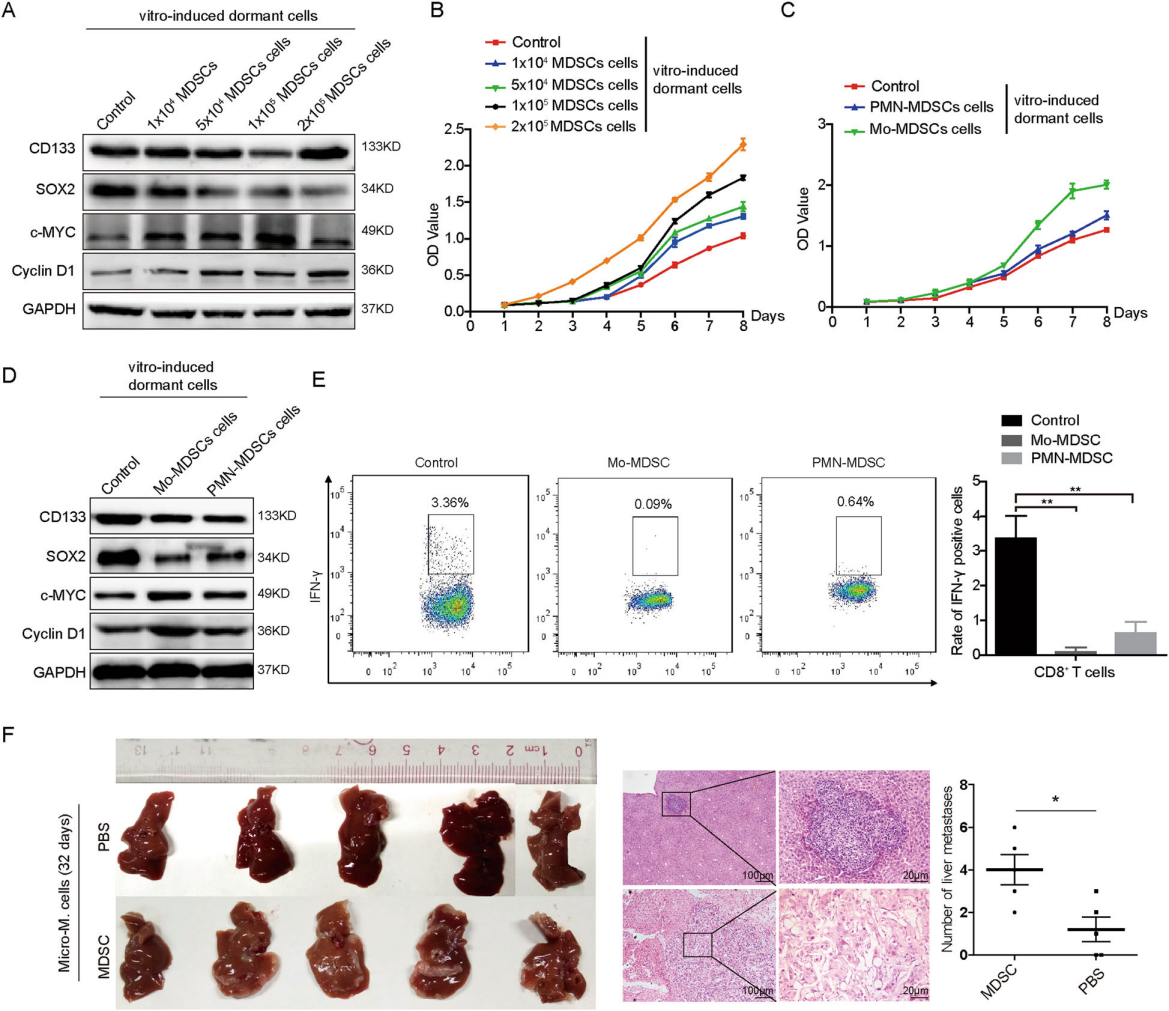
MDSCs activate dormant cell growth. **A** CD133, SOX2, c-Myc, and Cyclin D1 protein expressions in micro-metastatic cells or vitro-induced-dormant cell co-cultured with different numbers of MDSCs was detected by western blot. **B** Vitro-induced-dormant cells proliferation with different numbers of MDSCs co-culture were detected by CCK-8. **C** Vitro-induced-dormant cells proliferation with Mo-MDSCs or PMN-MDSCs co-culture were detected by CCK-8. **D** CD133, SOX2, c-Myc, and Cyclin D1 protein expressions in vitro-induced-dormant cells with Mo-MDSCs or PMN-MDSCs co-culture were detected by western blot. **E** IFN-γ in CD8^+^ T cells with Mo-MDSCs or PMN-MDSCs co-culture were detected by Flow cytometry. **F** Effect of Mo-MDSCs on promoting HT29 cells metastasis in vivo.

To test the effect of MDSCs on the activation of dormant cells, we constructed a tumor dormancy model in vitro by short-term chemotherapy^20^. We treated HT29 cells with 5-fluorouracil and oxaliplatin in a short time, and found that with the increasing of the concentration of chemicals, the expression of cyclin D1 and c-Myc of HT29 cells was decreased, while the levels of stemness marker CD133 and SOX2 were increased (Supplementary Fig. S2A). CCK-8 assays showed that the proliferation of HT29 cells treated with different concentrations of chemicals was sharply decreased (Supplementary Fig. S2B). However, high concentration (20 μM) of 5-fluorouracil and oxaliplatin-induced apoptosis of most of the HT29 cells and 10 μM of 5-fluorouracil and oxaliplatin was suitable for the following experiments. PMN-MDSCs (CD11b^+^Ly6G^+^Ly6C^−^) and Mo-MDSCs (CD11b^+^ Ly6C^high^ Ly6C^−^) were isolated from tumor metastases in mice and co-cultured with vitro-induced-dormant HT29 cells. Results of western blotting showed that MDSCs decreased the levels of CD133, SOX2, while increased the levels of cyclin D1, c-Myc in vitro-induced-dormant HT29 cells (Fig. 3A). The more of MDSCs was treated, the stronger effect of MDSCs on the proliferation of vitro-induced-dormant HT29 cells (Fig. 3B). Both Mo-MDSCs and PMN-MDSCs down-regulated CD133, SOX2 expression and up-regulated cyclin D1, c-Myc expression in vitro-induced-dormant HT29 cells. However, Mo-MDSCs induced the proliferation of vitro-induced-dormant HT29 cells more obviously than PMN-MDSCs (Fig. 3C, D). Meanwhile, we detected the function of MDSCs on the suppression of CD8^+^ T cells. Results showed that both PMN-MDSCs and Mo-MDSC obviously reduced the IFN-γ positive CD8^+^ T cells (Fig. 3E).

We also assessed the effect of MDSCs on the activation of dormant cells and metastatic outgrowth in vivo. We injected 5 × 10^6^ HT29 cells into cecum serosa and then 1 × 10^5^ MDSCs via tail vein in BALB/c nude mice. Results showed that compared to the control group, MDSCs obviously promoted liver macro-M and shortened the duration of liver micro-M of HT29 cells (Fig. 3F). The above data indicate that MDSCs play an important role in the reactivation of CRC dormant cells.

### CCL7 secreted by Mo-MDSCs is required for the reactivation of dormant cells

**Fig. 4:**
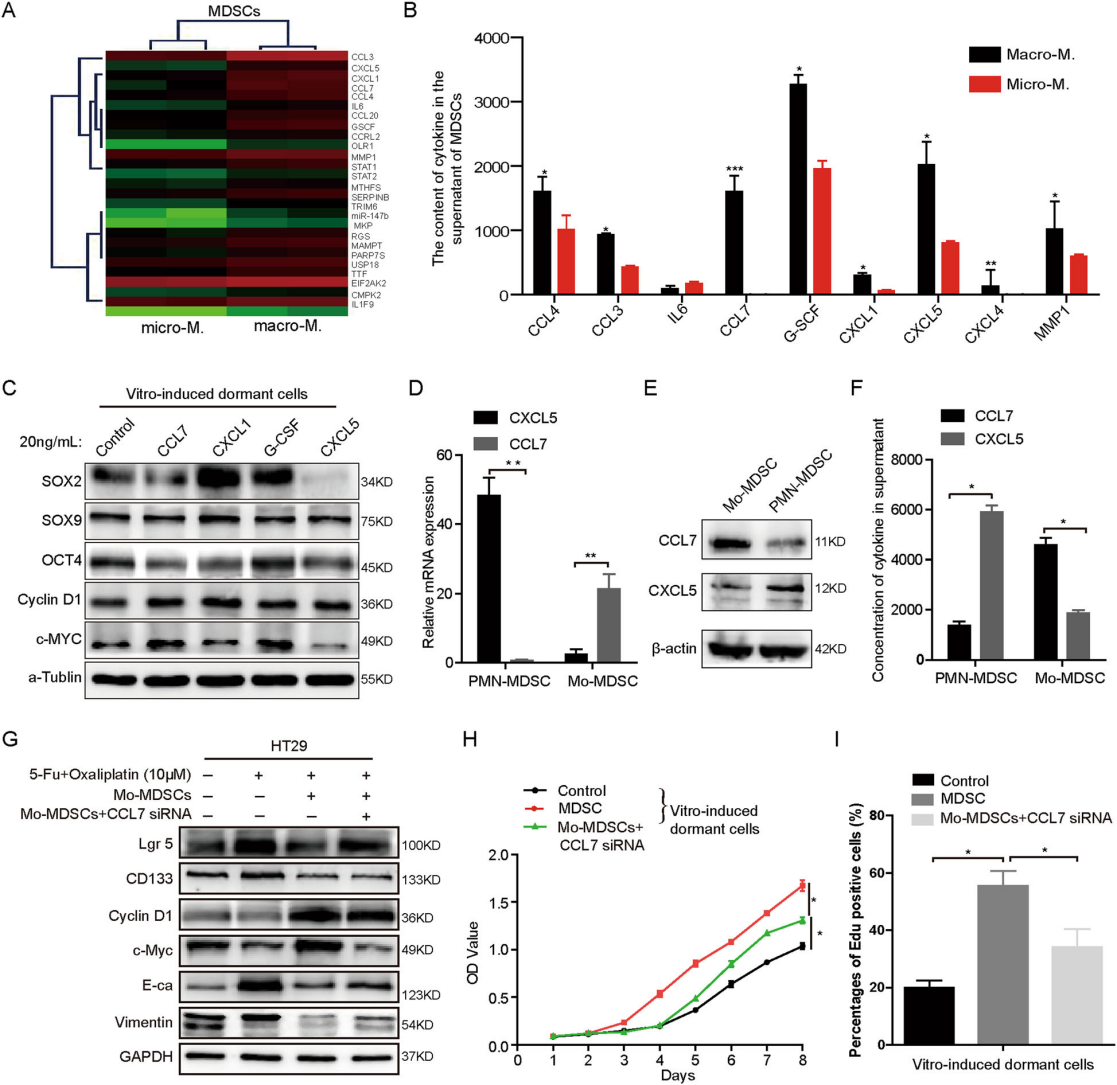
CCL7 secreted by Mo-MDSCs is required for dormant cell activation. **A** Gene array analyses of the MDSCs in micro-metastases and macro-metastases. **B** Cytokines in the cultural supernatant of mice macro-metastases and micro-metastases MDSCs were detected by Elisa assays. **C** The protein levels of c-Myc, Cyclin D1, OCT4, SOX9, and SOX2 in vitro-induced-dormant cells stimulated with CXCL5, CCL7, G-CSF, CXCL5, and CXCL1 (20 ng/mL) detected by western blotting. **D**, **E** The mRNA and protein of CCL7 in Mo-MDSCs and PMN-MDSCs were detected by Q-PCR and western blotting. **F** The contents of CCL7 in the cultural supernatant of Mo-MDSCs or PMN-MDSCs detected by Elisa assays respectively. **G** The protein levels of vimentin, E-ca, Cyclin D1, c-Myc, CD133, and Lgr5 in vitro-induced-dormant cells co-cultured with Mo-MDSCs or Mo-MDSCs siRNA. **H** Effect of vitro-induced-dormant cells co-cultured with Mo-MDSCs or Mo-MDSCs siRNA on cell proliferation detected by CCK-8 assay. **I** Effect of vitro-induced-dormant cells co-cultured with Mo-MDSCs or Mo-MDSCs siRNA detected by EdU assay. Error bars represent mean ± SD from at least three independent experiments. **P < 0.01, *P < 0.05.

To explore the potential mechanism of MDSCs governing tumor dormancy, we isolated MDSCs from liver micro-M and macro-M of CRC in mice and then performed trace RNA sequencing (RNA-seq, Fig. 4A). Based on the RNA-seq data, we selected the top 9 differentially expressed cytokines and detected them in MDSCs supernatant (Fig. 4B). ELISA results showed that the content of four factors CXCL1, CXCL5, G-CSF, CCL7 in the supernatant of MDSCs was the most significant differences. Then, vitro-induced-dormant HT29 cells were treated with recombinant proteins of four cytokines, with the results that only CCL7 could up-regulate the expression of Cyclin D1, c-Myc, and down-regulate SOX2, OCT4 expression (Fig. 4C). Then, we detected the level of CCL7 in PMN-MDSCs and Mo-MDSCs or their supernatants. The results of real-time PCR and Western blot showed that the expression of CCL7 in Mo-MDSC was much higher than that in PMN-MDSC (Fig. 4D, E). ELISA results confirmed the higher concentration of CCL7 in Mo-MDSC supernatant (Fig. 4F). In the tumor local site, Mo-MDSCs are consistently shown to have stronger suppressive activity than PMN-MDSCs^21^. Thus, we focused on CCL7 secreted by Mo-MDSCs for further study. We co-cultured vitro-induced-dormant HT29 cells with Mo-MDSCs transfected with CCL7 siRNA. Results of Western blot showed that compared to Mo-MDSCs/NC group, CCL7 knockdown decreased the level of c-Myc, increased the expressions of SOX2 and Lgr5 (Fig. 4G). Results of CCK-8 and EdU assays also validated that CCL7 knockdown at least partially reversed the promotion of the proliferation of dormant HT29 cells induced by MDSCs (Fig. 4H, I and Supplementary Fig. S3A). What’s more, we also explore the effects of CCL7 on the migration and invasion of vitro-induced-dormant cells. Results of in vitro cell scratch assays showed that CCL7 inhibition had slight effects on the migrations of vitro-induced-dormant cells (Supplementary Fig. S3B). While transwell assays presented that CCL7 knockdown had no significant effects on the invasion of vitro-induced-dormant cells (Supplementary Fig. S3C). These data indicate that Mo-MDSCs mainly promote the rapid growth of CRC dormant cells via secreting CCL7.

### CCL7 promotes the reactivation of dormant cells by regulating the JAK-STAT3 pathway

**Fig. 5:**
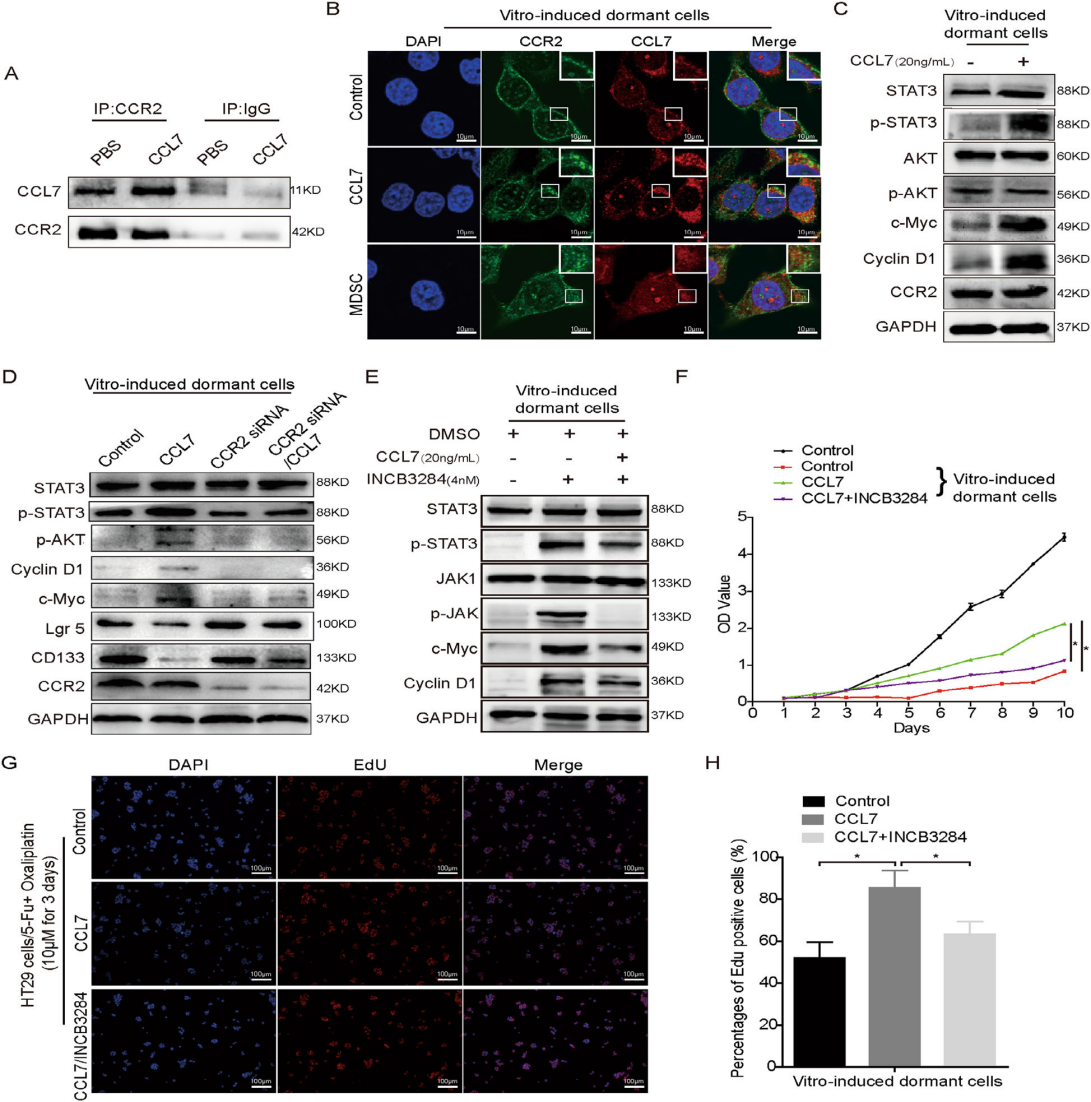
CCL7 stimulates the JAK-STAT3 pathway to increase MDSCs accumulation. **A** The combinations of CCR2 with STAT3, JAK, and CCL7 were detected by CO-IP. **B** The combination of CCL7 and CCR2 on the surface of dormant cancer cells by immunofluorescence. **C** The protein levels of CCR2, p-AKT, AKT, p-STAT3, STAT3, Cyclin D1, and c-Myc in vitro-induced-dormant cells treated with CCL7 (20 ng/mL). **D** The protein levels of CCR2, p-AKT, p-STAT3, STAT3, Cyclin D1, c-Myc, CD133, and Lgr5 in vitro-induced-dormant cells treated with CCL7 or CCR2 siRNA. **E** The protein levels of p-JAK, p-STAT3, STAT3, JAK, Cyclin D1, and c-Myc in vitro-induced-dormant cells treated with CCL7 or CCR2 antagonist INCB3284. **F** Cell proliferation of Vitro-induced-dormant cells treated with CCL7 or CCR2 antagonist INCB3284 was detected by CCK-8. **G**, **H** Cell proliferation of vitro-induced-dormant cells treated with CCL7 or CCR2 antagonist INCB3284 was detected by EdU assay. Error bars represent mean ± SD from at least three independent experiments. **P < 0.01, *P < 0.05.

Previous studies have reported that CCL7 can combine with CCR2 to activate the STAT3 signal pathway and thus promote tumor cell metastasis^22–24^. We examined whether Mo-MDSCs secreted CCL7 binds to the CCR2 receptor of dormant HT29 cells. CoIP analyses showed that CCL7 could physically bind to its receptor CCR2 of dormant HT29 cells and treatment of recombinant protein CCL7 reduced the interaction between CCR2 and STAT3 or JAK (Fig. 5A). Results of IF also showed that CCL7 and CCR2 had obvious co-localization (Fig. 5B). Treatment of dormant HT29 cells with human recombinant protein CCL7 increased the phosphorylation of STAT3 and the levels of c-myc and cyclin D1, decreased the levels of Lgr5 and CD133, but the expression of total STAT3 and CCR2 did not change significantly (Fig. 5C, D). Silencing CCR2 in dormant HT29 cells resulted in the opposite results of CCL7 treatment. However, there was no significant change in p-STAT3, c-myc, cyclin D1, Lgr5, and CD133 after the reintroduction of CCL7 in HT29 depleting CCR2 cells (Fig. 5D). Moreover, CCR2 antagonist INCB3284 (4 nM) effectively blocked the activation of the JAK-STAT3 pathway and upregulation of c-Myc and cyclin D1 induced by CCL7 (Fig. 5E). Results of CCK-8 and EdU assays proved that CCL7 promoted the proliferation of vitro-induced-dormant HT29 cells, while CCR2 antagonist reversed the effect induced by CCL7 (Fig. 5F–H). These results demonstrate that CCL7 binds to CCR2 of dormant cells and then promotes their outgrowth by activating the JAK-STAT3 pathway.

### CCL7 inhibitor effectively prevents CRC metastasis and prolongs the survival of mice

**Fig. 6:**
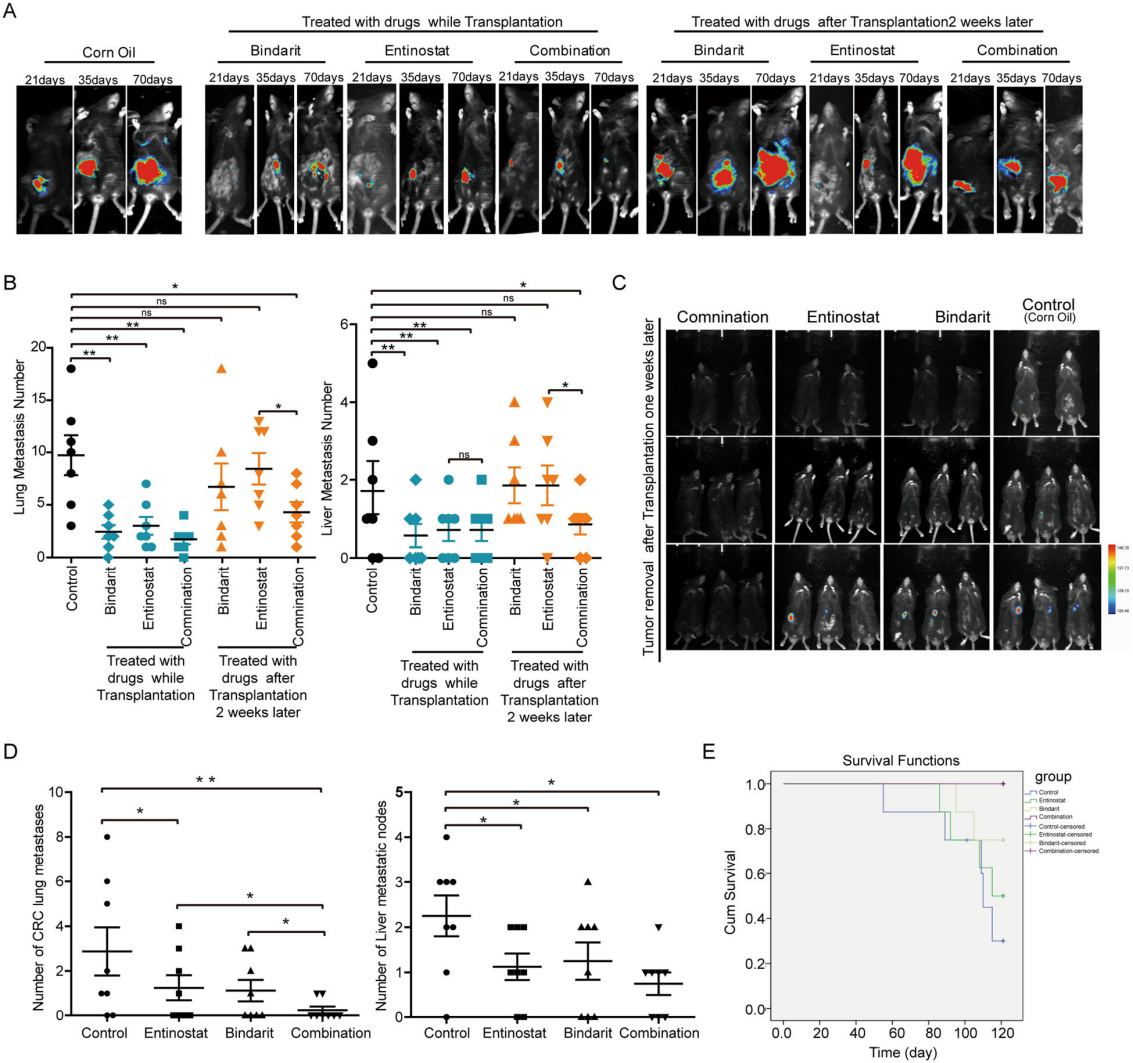
CCL7 inhibitor reduced CRC macro-metastases and prolonged survival in mice. **A** Effect of CCL7 inhibitor Bindarit and MDSC inhibitor Entinostat on the CRC metastasis was observed by Bruber In Vivo MS FX Pro Imager. **B** Quantities of the metastases nodes in CRC mice were observed by light microscope. **C** The receiving primary tumor resection CRC mice were treated with CCL7 inhibitor Bindarit or MDSC inhibitor Entinostat, and their metastases were observed by Bruber In Vivo MS FX Pro Imager. **D** Quantities of the nodes of metastases in CRC mice treated with primary tumor resection and observed by light microscope. **E** The survival of mice treated with CCL7 inhibitor Bindarit and MDSC inhibitor Entinostat by Kaplan–Meier analysis.

We then investigated the therapeutic potential of CCL7 inhibitor on CRC metastasis. We first implanted CMT-93 cells labeled with luciferase in the cecal serosa of C57 mice, and gave them two different treatments. The first treatment was to give Bindarit (CCL7 inhibior, 5 mg/kg) or Entinostat (MDSCs inhibitor, 10 mg/kg)^25^ or a combination of Bindarit and Entinostat by gavage twice a week for 2 months at the time of tumor implantation, the other was to give these drugs 2 weeks later after tumor transplantation. Then the liver metastases were observed by Caliper IVIS Lumina II once a week for 4 months. The results showed that administration of Bindarit or Entinostat or both during tumor implantation could significantly inhibit lung and liver metastasis of CRC. However, administration of Bindarit or Entinostat alone 2 weeks after implantation had no effect on lung and liver metastasis of CRC, while the combination of Bindarit and Entinostat could inhibit liver and lung metastasis (Fig. 6A, B and Supplementary Fig. S4A). Next, we implanted CMT-93 cells in the cecal serosa of C57 mice and resected the primary tumor 1 week later, following with the drug treatment. Treatment of Bindarit or Entinostat or both significantly suppressed lung and liver metastases and improved the survival of the mice (Fig. 6C–E and Supplementary Fig. S4B, *P* < 0.05). Moreover, the combination of Bindarit and Entinostat resulted in less lung and liver metastatic nodes than Bindarit or Entinostat alone, while it had little effect on the survival of mice compared with Bindarit or Entinostat alone (Fig. 6C–E and Supplementary Fig. S4B). These results suggest that preventive administration of CCL7 and MDSCs inhibitors can significantly inhibit CRC cell proliferation and metastasis or reduced tumor recurrence after radical operation.

The level of CCL7 in serum of CRC patients is highly linked with the short-time recurrence and distant metastasis.

**Fig. 7:**
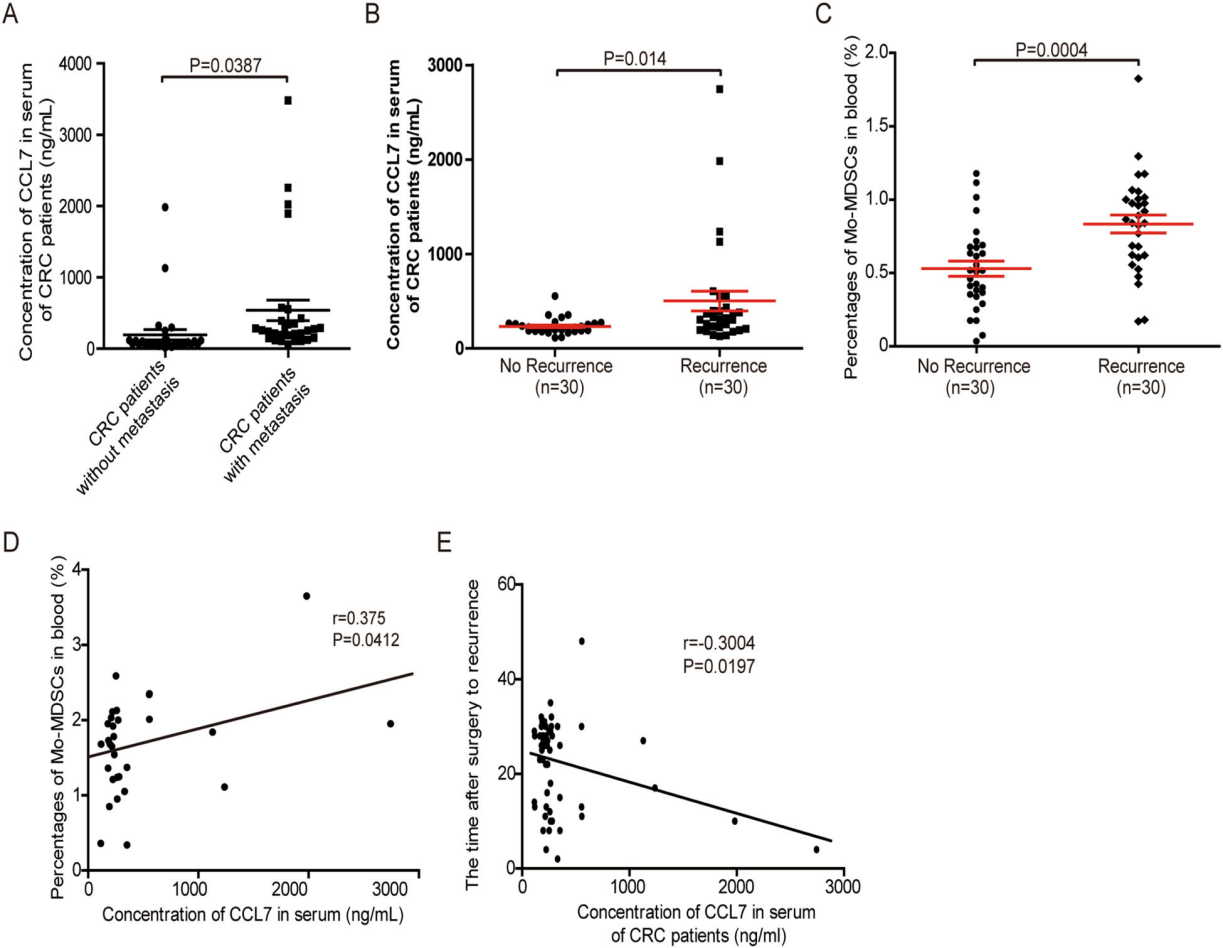
The level of CCL7 in serum of CRC patients is highly linked with the short-time recurrence and distant metastasis. **A** The level of CCL7 in the serum of CRC patients with or without metastasis detected by Elisa assays (*n* = 60). **B** The level of CCL7 in the serum of CRC patients with or without short-time recurrence (<2 years) detected by Elisa assays (*n* = 30). **C** The percentages of MDSCs in the blood of CRC patients with or without short-time recurrence (<2 years) detected by Flow cytometry (*n* = 30). **D** Relationship of the level of CCL7 in the serum with the percentages of MDSCs in the blood of CRC patients (*n* = 30). **E** Relationship of the level of CCL7 in the serum with the time after surgery to recurrence (*n* = 60).

Finally, we analyzed the level of CCL7 and the number of Mo-MDSC in peripheral blood of CRC patients. The results showed that the level of CCL7 in serum was significantly higher in CRC patients with tumor metastasis or short-term recurrence than those without metastasis or recurrence (Fig. 7A, B). The number of Mo-MDSCs in the blood of CRC patients was also increased in CRC patients with short-term recurrence compared with those without recurrence (Fig. 7C). There was a positive correlation between the level of CCL7 and the number of Mo-MDSCs in the blood of CRC patients (Fig. 7D). And the level of CCL7 in the serum of CRC patients was negatively related to recurrence after radical surgery (Fig. 7E). These data demonstrate that CCL7 might be a promising marker for CRC patients to predict the risk of recurrence and metastasis.

## Discussion

After removal of the primary tumor, metastatic tumor cells may undergo a long period marked by the absence of clinical symptoms (micrometastasis). We define this period “tumor latency” which reveals the capacity of DTCs and/or micro-metastases^26^. Unfortunately, micro-metastatic cells will eventually produce overt metastasis, which is the primary cause of death of tumor patients and current treatments fail to provide durable responses. There are currently no effective interventions that prevent the metastatic progression from latency. This study explores the role of MDSCs, especially Mo-MDSCs in the metastatic progression of CRC. Certain DTCs remain dormant for an extended period, which is determined by the lack of proliferating markers (Ki67, c-Myc, cyclin D1) and may be responsible for conventional chemoradiotherapy resistance^27^. In the process of tumor cell dormancy, DTCs may retain stem-like properties such as quiescence, with the increased expression of stem cell markers CD44, SOX2, CD133, or Lgr5^28,29^. The transient EMT/MET gradient state linked to CSC-like traits may dictate whether DTCs will remain dormant or emerge to metastatic outgrowth. As the EMT/MET state progressively tends to a more epithelial phenotype, this, in turn, will increase cell proliferation and differentiation of the growing macro-metastases^30^. Based on the above studies, we chose HT29 cells with dormant characteristics to constructed the CRC metastatic latency model in BALB/c nude mice and observed the latent stage in metastatic models of CRC by using CMT293 cells in C57BL/6 mice with primary tumor was removed or not. Expectedly, the results showed that liver micro-M in all mice models contained dormant cells. The duration of liver micro-M could be prolonged by resection of primary focus.

MDSCs are inherent immature cells in the tumor immune microenvironment (TIME) and expand in the tumor site, lymphoid tissues, and peripheral blood during inflammation, infection, and cancer^31^. There are two major subsets of MDSC in both mice and humans: polymorphonuclear (PMN)-MDSC and monocytic (Mo)-MDSC. PMN-MDSCs share many morphological and phenotypic characteristics of neutrophils, whereas M-MDSCs are similar to monocytes^32^. Ample evidence supports a close association between MDSC accumulation and clinical outcome in cancer patients^33^ and the increase in the immunosuppressive potential of MDSCs was correlated with lymph node metastasis in breast cancer patients^34^. We analyzed the immune profiles between liver micro-M and macro-M in C57BL/6 mice and found that MDSC is one of the fastest-growing immune cells in mice. And the increase of Mo-MDSCs was more obvious than that of PMN-MDSC from liver micro-M to macro-M. Till now, the effect of MDSCs on tumor dormancy keeps unknown. We constructed the CRC dormancy model in vitro by short-time chemotherapy of HT29 cells. When MDSCs were co-cultured with the vitro-induced-dormant HT29 cells, the proliferation of dormant HT29 cells was increased. Moreover, Mo-MDSCs induced the proliferation of dormant HT29 cells more obviously than PMN-MDSCs. Results of in vivo assays also confirmed that MDSCs obviously promoted liver macro-M and shortened the duration of liver micro-M of HT29 cells. Some evidence shows that PMN-MDSCs play a major role in the regulation of tumor-specific T-cell tolerance in peripheral blood and lymphoid organs. However, in the tumor local site, Mo-MDSCs are consistently shown to have stronger suppressive activity than PMN-MDSCs^31^. Our results show that MDSCs, especially Mo-MDSCs play an important role in the reactivation of CRC dormant cells.

During the exploration of the underlying mechanisms of MDSCs involved in the regulation of CRC dormancy, we found that CCL7 secreted by Mo-MDSCs acted as an important role in the promotion of the proliferation of dormant HT29 cells induced by MDSCs. CCL7, a member of the CC subfamily, is widely expressed in various types of cells including monocytes fibroblasts, intestinal epithelial cells, and some malignant tumors^35^. CCL7 can combine with CCR1, CCR2, and other receptors on immune cells, and exert antitumor effect by recruiting T-lymphocytes, natural killer cells, dendritic cells, and other immune cells^36,37^. Androgen receptor knockdown in prostate cancer promotes cancer cell migration/invasion via CCL2- dependent STAT3 activation and EMT pathways^24^. Activated STAT3 signaling can regulate a variety of downstream target genes such as VEGF, BCL-xl, c-myc, and cyclin D1 to induce angiogenesis, inhibit apoptosis and promote cell proliferation^38^. Although several previous articles have been reported that CCL7 can combine with CCR2 to activate the STAT3 signal pathway and thus promote tumor cell metastasis, we firstly found that CCL7 secreted by MDSCs played an important role in the activation of dormant tumor cells during the distant metastasis stage through CCL7/CCR2/STATS pathway.

Accumulating data indicate that metastatic dissemination often occurs early during tumor formation^39^. However, current tumor therapies, whether surgery or radiotherapy, chemotherapy, immunotherapy, and other combined treatments, can not completely eradicate the metastatic tumor cells. Therefore, how to maintain the metastatic tumor cells in a long-term dormancy or no recurrence and proliferation state is another new treatment strategy. In the metastatic model of C57 mice, our results showed that the long-term treatment of low-dose Bindarit or Entinostator both by gavage at the time of tumor implantation, rather than the treatment of drugs 2 weeks after tumor implantation, could significantly inhibit lung and liver metastasis of CRC. When the primary tumor was removed a week later after tumor implantation, the administration of Bindarit or Entinostat or both significantly suppressed lung and liver metastases and improved the survival of the mice. Our data suggest that long-time and low-dose of CCL7 and/or MDSC inhibitors treatment notably reduced the recurrence and metastasis and prolong the survival time of mice after radical surgery. It may provide a novel strategy to prevent metastasis in CRC patients.

Finally, we explored the clinical correlation of Mo-MDSCs with CCL7 level in the blood of CRC patients. The results showed that the level of CCL7 in serum was significantly higher in CRC patients with tumor metastasis or short-term recurrence than those without metastasis or recurrence. The number of Mo-MDSC in blood was positively related to the levels of CCL7 in serum of CRC patients. These data further proved that CCL7 might be a promising marker for CRC patients to predict the risk of recurrence and metastasis.

## Conclusions

In summary, our study highlights the novel role and regulatory mechanisms of Mo-MDSC in reactivating dormant cells. CCL7 secreted by Mo-MDSCs binds to CCR2 of dormant cells and promotes the transition from tumor dormancy to metastatic outgrowth through the JAK-STAT3 signaling pathway. Long-time and low-dose of CCL7 inhibitor treatment might be a promising alternative strategy to induce tumor dormancy and immune evasion, thus reduce tumor recurrence and prolong the survival of CRC patients.

## Supplementary information

Supplementary materials

## Data Availability

The data sets used and/or analyzed during the current study are available from the corresponding author on reasonable request.
