# Supplementary material for: Inhibition of CCL7 derived from Mo-MDSCs prevents metastatic progression from latency in colorectal cancer
Source: Cell Death Dis. 2021 May 13;12(5):484. doi: 10.1038/s41419-021-03698-5 (PMC8119947; doi:10.1038/s41419-021-03698-5)
Supplement: Supplementary file 1 — Supplementary materials [file 41419_2021_3698_MOESM1_ESM.docx]

**Supplemental materials**

**Supplementary Tables**

**Supplementary Table 1.** Realtime Q-PCR primers shown in supplementary Table 1**.**

| Mouse gene | Forward primer (5' to 3) | Reverse primer (5' to 3) |
| --- | --- | --- |
| CCL4 | TTCCTGCTGTTTCTCTTACACCT | CTGTCTGCCTCTTTTGGTCAG |
| MMP1 | AACTACATTTAGGGGAGAGGTGT | GCAGCGTCAAGTTTAACTGAA |
| GAPDH | AATGGATTTGGACGCATTGGT | TTTGCACTGGTACGTGTTGAT |
| CXCL5 | GTTCCATCTCGCCATTCATGC | GCGGCTATGACTGAGGAAGG |
| CCL7 | CCACATGCTGCTATGTCAAGA | ACACCGACTACTGGTGATCCT |
| CXCL1 | CTGGGATTCACCTCAAGAACATC | CAGGGTCAAGGCAAGCCTC |
| CXCL4 | GTCCCGAAGAAAGCGATGGA | TATAGGGGTGCTTGCCGGTC |
| G-CSF | TGCTTAAGTCCCTGGAGCAAG | CCACTGTGGAGCTGGCTTAG |
| IL-6 | GGCGGATCGGATGTTGTGAT | GGACCCCAGACAATCGGTTG |
| CCL3 | TTCTCTGTACCATGACACTCTGC | CGTGGAATCTTCCGGCTGTAG |

**Supplementary Table 2.** Antibody list for western blotting was shown in Supplementary Table 2.

| Antibody | Specificities | Source | Dilution | Catalog |
| --- | --- | --- | --- | --- |
| GAPDH | human, mouse, rat | Mouse Monoclonal | 1:1000 | Proteintech #60004 |
| α-Tublin | human, mouse, rat | Rabbit Polyclonal | 1:1000 | Proteintech #11224 |
| CD133 | human | Mouse Monoclonal | 1:300 | Proteintech #66666 |
| SOX2 | human, mouse, rat | Rabbit Polyclonal | 1:500 | Proteintech #11064 |
| c-myc | human, mouse, rat | Rabbit Polyclonal | 1:300 | Proteintech # 10828 |
| Cyclin D1 | human, mouse, rat | Rabbit monoclonal | 1:300 | ab16663 |
| OCT4 | Human, mouse | Rabbit Polyclonal | 1:300 | Proteintech #11263 |
| CCR2 | human, rat | Rabbit Polyclonal | 1:500 | Proteintech #16153 |
| STAT3 | mouse, rat, human | Mouse monoclonal | 1:500 | CST #9139 |
| p-STAT3 (Tyr705) | human, mouse, rat | Rabbit Polyclonal | 1:500 | CST #9131 |
| Lgr5 | mouse, rat, human | Rabbit monoclonal | 1:500 | ab75850 |
| SOX9 | human, mouse, rat | Rabbit monoclonal | 1:500 | ab185966 |
| JAK1 | human, mouse | Rabbit Polyclonal | 1:1000 | CST #3332 |
| p-JAK1/2 | human, mouse | Mouse monoclonal | 1:500 | CST #66245 |
| E-cadherin | human, pig | Mouse monoclonal | 1:500 | Proteintech # 60335 |
| vimentin | human, mouse, rat | Rabbit Polyclonal | 1:1000 | Proteintech #10366- |
| AKT | human, mouse, rat | Mouse monoclonal | 1:1000 | CST #2920 |
| p-AKT (Ser473) | Human, mouse, rat | Rabbit monoclonal | 1:500 | CST #4060 |

**Supplementary Table 3.** The sequences of genes’ shRNAs were shown in supplementary Table 3.

| Genes’ siRNA | Target sequence |
| --- | --- |
| Mouse CCL7 siRNA1 | GCTTTCAGCATCCAAGTGT |
| Mouse CCL7 siRNA2 | CCACATGCTGCTATGTCAA |
| Mouse CCL7 siRNA3 | CCTGGGAAGCTGTTATCTT |
| Human CCR2 siRNA1 | TGTATCACATCGGTTTATTT |
| Human CCR2 siRNA2 | CACATCTCGTTTCTCGGTTT |
| Human CCR2 siRNA3 | TACCAACGAGAGCGGTGAA |

**Supplementary figures:**

Figure S1.


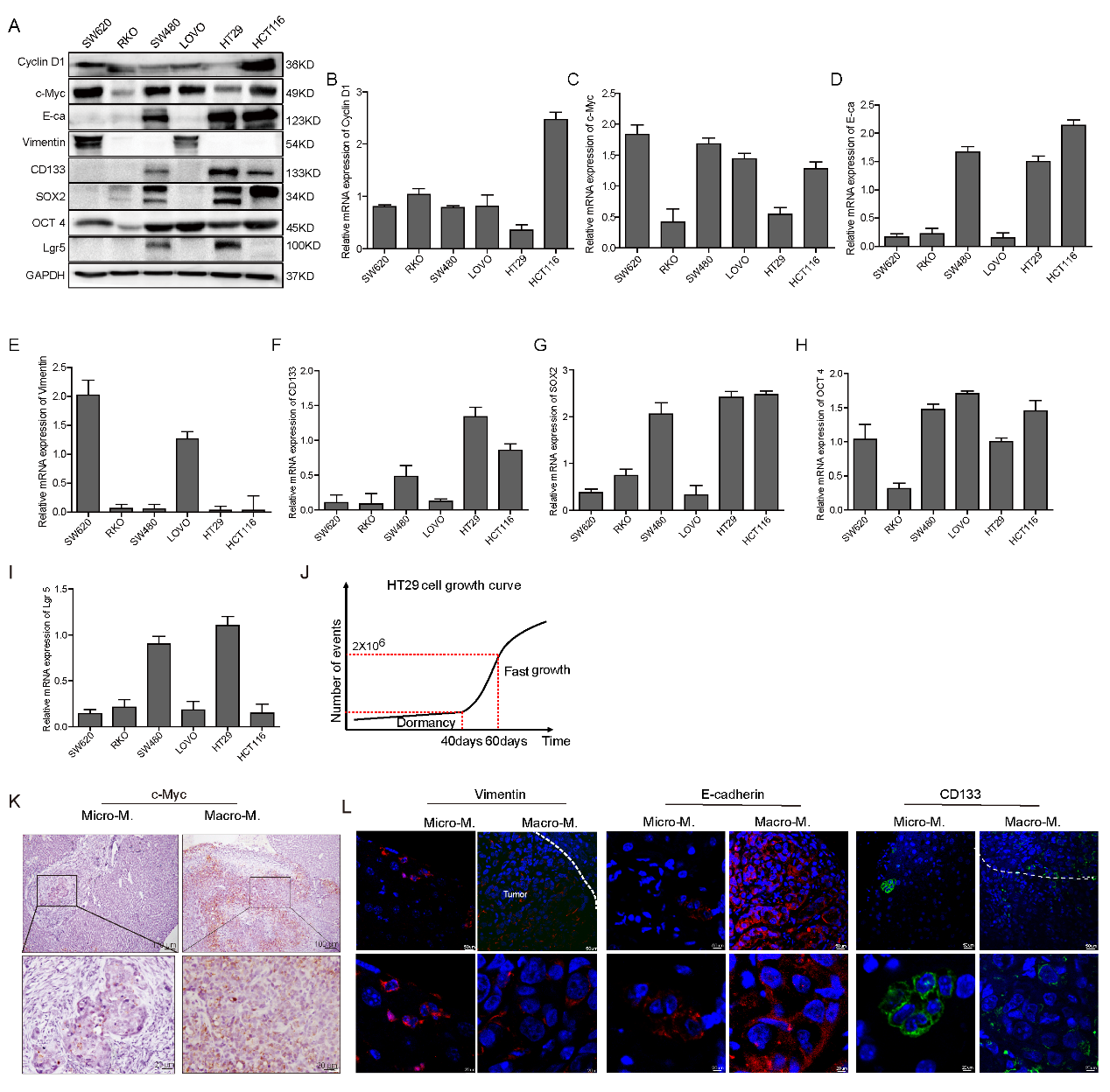


Figure S2.


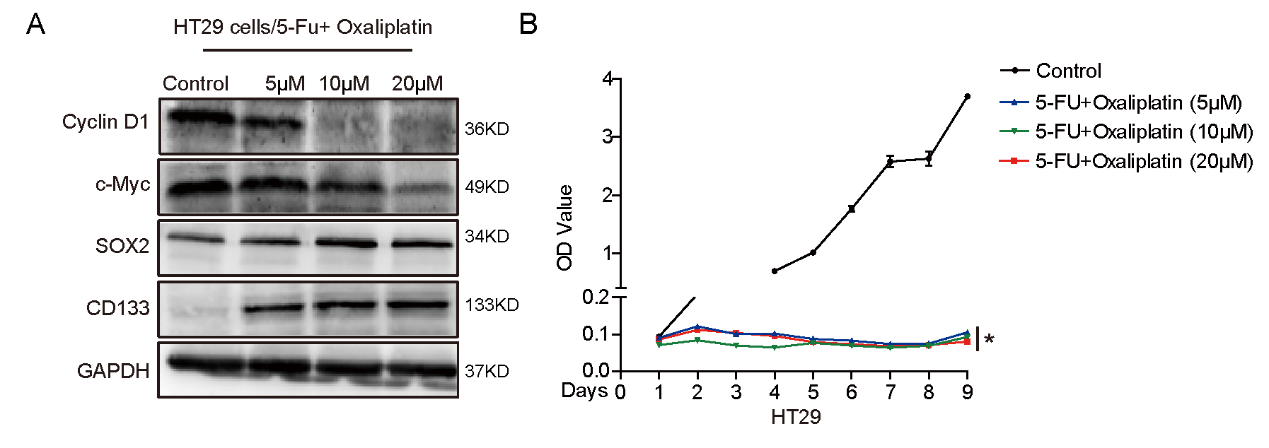


Figure S3


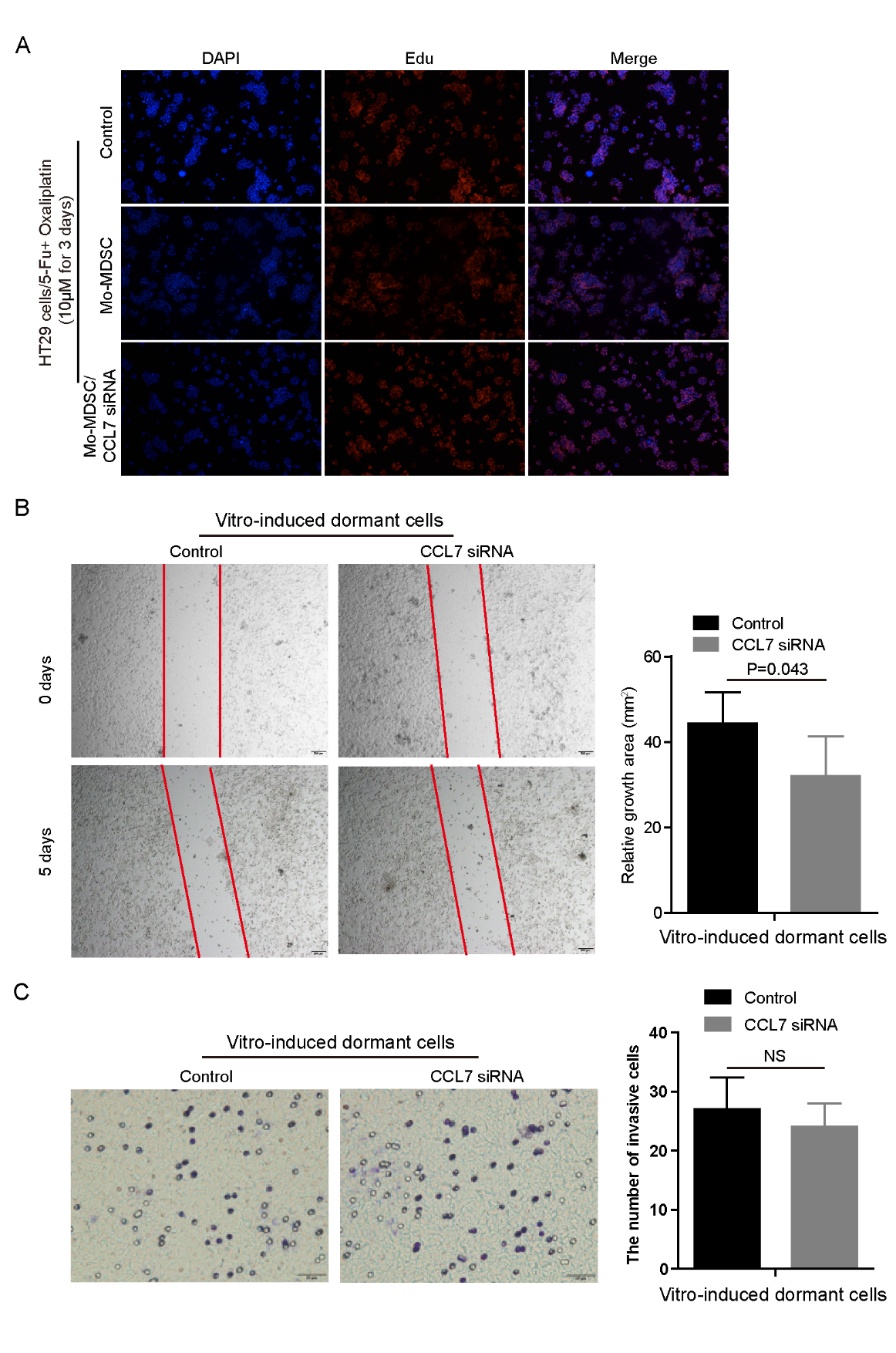


Figure S4.


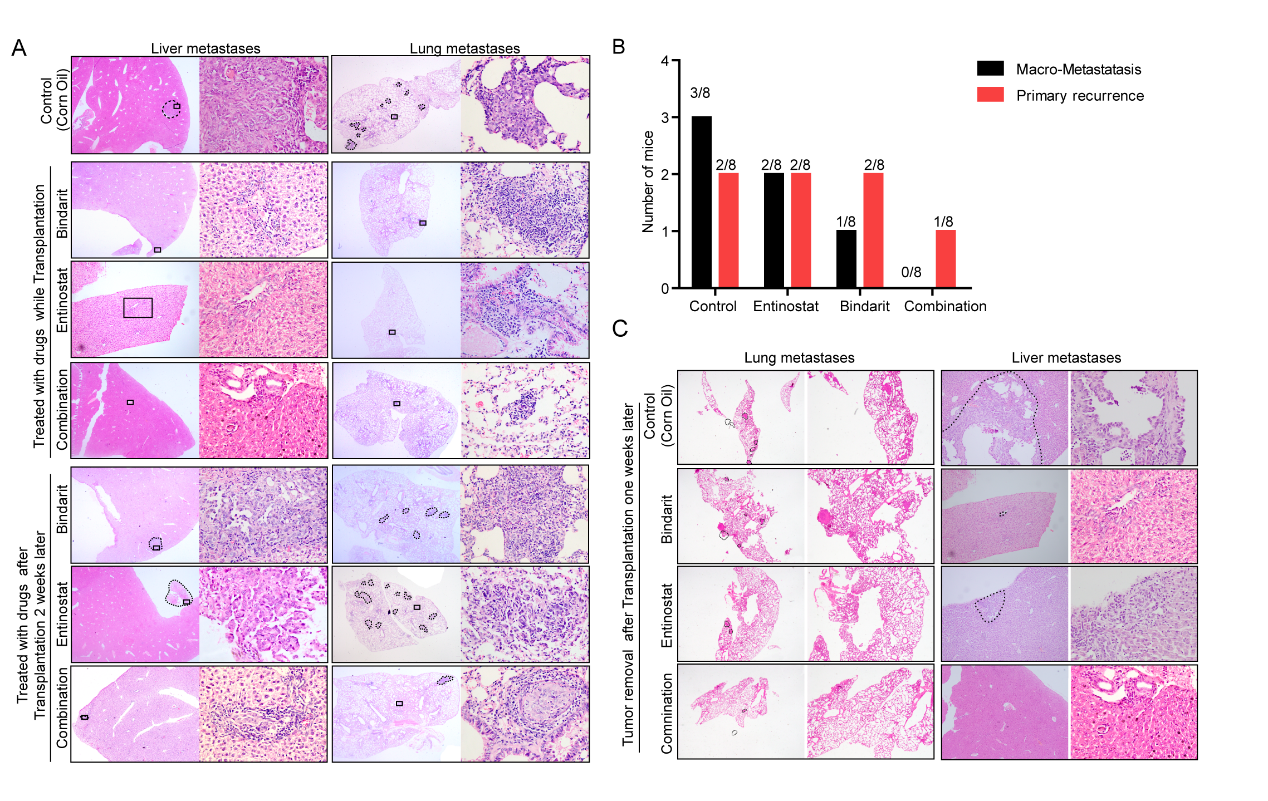


**Supplementary figure legends**

**Figure S1** (A) The protein levels of Cyclin D1, Lgr5, E-ca, CD133, c-Myc, SOX2, Vimentin and OCT 4 in six CRC cell lines. (B-I) The mRNA levels of Cyclin D1, Lgr5, E-ca, CD133, c-Myc, SOX2, Vimentin and OCT 4 in six CRC cell lines. (J) Intrahepatic growth curve of HT29 cells in liver of nude mice. (K) The immunohistochemistry stain of c-Myc in liver metastasis of nude mice by injection with HT29 cells. (L)The Immunofluorescence stainings of Vimentin, E-ca and CD133 in liver metastasis of nude mice by injected with HT29 cells. (M) The protein levels of CD133, SOX2, c-Myc, cyclin D1, Vimentin and E-cadherin in micrometastases and macrometastases. Tublin as internal parameter.

**Figure S2.** (A) The protein level of CD133, SOX2, c-Myc and cyclin D1 in HT29 cells treated with different concentrations of 5-fluorouracil and oxaliplatin. GAPDH as internal parameter. (B) Effect of different concentrations of 5-fluorouracil and oxaliplatin on cell proliferation of HT29 cells detected by CCK-8 assay.

**Figure S3.** (A) Effect of vitro-induced dormant cells co-cultured with Mo-MDSCs or Mo-MDSCs/CCL7 siRNA detected by Edu assays. (B-C) Effect of vitro-induced dormant cells co-cultured withCCL7 siRNA or not detected by in vitro cell scratch assay and transwell assays.

**Figure S4** (A) Effect of CCL7 inhibitor Bindarit and MDSC inhibitor Entinostat CRC on the metastasis of CRC mice without primary tumor resection observed by HE staining. (B) Quantities of metastatic mice treated with primary tumor resection and observed by Bruber In Vivo MS FX Pro Imager. (C) Effect of CCL7 inhibitor Bindarit and MDSC inhibitor Entinostat CRC on the metastasis of CRC mice treated with primary tumor resection and observed by HE staining.
